# Detailed Molecular and Structural Analysis of Dual Emitter IrQ(ppy)_2_ Complex

**DOI:** 10.3390/ma13071617

**Published:** 2020-04-01

**Authors:** Iulia Corina Ciobotaru, Daniel Nicolae Crisan, Primoz Šket, Constantin Claudiu Ciobotaru, Silviu Polosan

**Affiliations:** 1 National Institute of Materials Physics, Atomistilor 405A, 077125 Magurele, Romania; corina.ciobotaru@infim.ro (I.C.C.); daniel.crisan@infim.ro (D.N.C.); 2 Slovenian NMR Center, National Institute of Chemistry, Hajdrihova 19, Ljubljana SI-1000, Slovenia; primoz.sket@ki.si

**Keywords:** carbon analysis, organometallic compound, 2D NMR, EXAFS, molecular structure

## Abstract

The molecular structure of the 8-hydroxyquinoline–bis (2-phenylpyridyl) iridium (IrQ(ppy)_2_) dual emitter organometallic compound is determined based on detailed 1D and 2D nuclear magnetic resonance (NMR), to identify metal-ligands coordination, isomerization and chemical yield of the desired compound. Meanwhile, the extended X-ray absorption fine structure (EXAFS) was used to determine the interatomic distances around the iridium ion. From the NMR results, this compound IrQ(ppy)_2_ exhibits a *trans* isomerization with a distribution of coordinated N-atoms in a similar way to facial Ir(ppy)_3_. The EXAFS measurements confirm the structural model of the IrQ(ppy)_2_ compound where the oxygen atoms from the quinoline ligands induce the splitting of the next-nearest neighboring C in the second shell of the Ir^3+^ ions. The high-performance liquid chromatography (HPLC), as a part of the detailed molecular analysis, confirms the purity of the desired IrQ(ppy)_2_ organometallic compound as being more than 95%, together with the progress of the chemical reactions towards the final compound. The theoretical model of the IrQ(ppy)_2_, concerning the expected bond lengths, is compared with the structural model from the EXAFS and XRD measurements.

## 1. Introduction

The use of organometallic compounds in new technologies such as organic light-emitting diodes (OLED) [1,2,3,4,5,6,7,8], photovoltaic applications [9] or catalysis [10], requires different approaches during the chemical synthesis of these compounds, going from the photoluminescence, electroluminescence and internal quantum efficiency to the charge transport and the amorphous-crystalline interplay between these organometallic molecules [11,12,13,14,15,16,17,18].

Starting with classical monoligand compounds containing quinoline (q) and phenylpyridine (ppy) derivatives, such as Alq_3_ and Ir(ppy)_3_, which give single color electroluminescence [1,19], the architecture of these organometallics evolves towards more complex structures. These structures enable multicolor electroluminescence such as in the case of 8-hydroxyquinoline–bis (2-phenylpyridyl) iridium, known as IrQ(ppy)_2_, with a general formula C_31_H_22_IrN_3_O, or better external quantum efficiency in the OLED structures.

Depending on the desired organometallic compound, the chemical synthesis requires one, two or multiple reaction steps, following the Nogoyama procedure [20,21,22,23,24]. Generally, these syntheses are characterized by incomplete reactions and the formation of by-products. Therefore, the resulting compounds could form different molecular configurations close or far from the targeted structure. For example, in the case of IrQ(ppy)_2_ the chemical synthesis is done in two steps in which the [(C^N)_2_Ir-µ-Cl]_2_ intermediate compound from the first step reaction with phenylpiridine ligands is used in the second step reaction with quinoline ligands. The second step could lead to three different structures like IrQ(ppy)_2_, IrQ_2_(ppy) or even IrQ_3_ with different photophysical properties. The first structure yields dual green-red electroluminescence [25], while the last one gives pure red color in the OLED structures [26]. The red electroluminescent OLED represents a special class due to their low bandgap, which can lead to non-radiative emissions [27,28,29].

High-performance liquid chromatography (HPLC) and nuclear magnetic resonance spectroscopy (NMR) are the best techniques used to investigate the progress of the chemical reactions, the final products and their purity.

Regarding organometallic compounds, NMR spectroscopy measurements allow the identification of parameters related to the metal–ligand coordination, isomerization or even the ratio between ligands. All of these parameters are closely related to each other and describe a way to obtain a pure compound [30,31,32,33]. Furthermore, NMR spectroscopy measurements can determine the chemical yield of these reactions through the assignment of the off-structure peaks [34]. 

Complementarily, the enhanced X-ray absorption fine structure (EXAFS) measurements allow identification of interatomic distances for a complete view of the molecular structure of the organometallic compounds. The Ir-L_3_ EXAFS absorptions allow us to accurately determine the metal environment in the Ir(III) organic complexes with efficient phosphorescence emission. Such knowledge enables the validation of the preparation stages of these compounds by comparison with the crystallographic data. The EXAFS measurements were already applied to other organometallic compounds like [IrCp*(OH)_2_(Me_2_-NHC)] (final compound from the second step of reaction, prepared from the bis-Cl intermediate compound; Me_2_-NHC = N-dimethylimidazolin-2-ylidene, Cp* = pentamethy-lcyclopentadienyl), used as a catalyst for water oxidation at the electrode surface [35]. This method allows us to investigate the oxidation processes of the iridium complex, possibly towards IrO_3_, which is also catalytically active [36], and/or the iridium coordination in different chemical compounds. 

This paper proposes a complete investigation by HPLC, NMR, EXAFS and XRD techniques, to identify the chemical structure of IrQ(ppy)_2_ organometallic compound. It is focused on the molecular structure, resulting from the chemical synthesis, the ratio between phenylpyridine and quinoline ligands, the modality of coordination (C^N), the photochemical isomerization (cis, trans). The bonding between the metal and ligands (Ir-N, Ir-C, Ir-O) was also determined and compared with the interatomic distances obtained from XRD of an existing crystallographic structure.

## 2. Materials and Methods 

The IrQ(ppy)_2_ synthesis implies a few additional steps which can influence the efficiency of the chemical reaction and the crystallization process of the obtained powder. For example, a faster crystallization from dichloromethane can reduce the physical oxidation of the organometallic compound but influences the isomerization and crystallography of the obtained powder. 

Ir(III) complex 8-hydroxyquinoline–bis (2-phenylpyridyl) iridium (IrQ(ppy)_2_) organometallic compound was chemically synthesized with two types of ligand: 2-phenylpyridine and quinoline, in a standard two-step reaction procedure, described previously [7]. Briefly, a mixture of iridium chloride hexahydrate and 2-phenylpyridine in 2-ethoxyethanol was refluxed in an argon atmosphere for 12 h at 150 °C. The yellow precipitate was cooled at room temperature and washed with ethanol and then dried in vacuum forming a [(C^N)_2_Ir-μ-Cl]_2_ bridged dimer. The reaction of the resulting dimer with 8-hydroxyquinoline, in the second step, led to the formation of the final complex, IrQ(ppy)_2_. The obtained mixture was heated to reflux under a nitrogen atmosphere for 8 h at 140 °C. The crude product, obtained after filtration, was dissolved in CH_2_Cl_2_ and concentrated in a vacuum. All chemicals were taken from Sigma Aldrich (St. Louis, MO, USA) and used without further purification.

Iridium coordination with the quinoline and phenylpiridine ligands in the IrQ(ppy)_2_ compound was investigated by Extended X-ray Absorption Fine Structure (EXAFS) spectroscopy using the Ir-L_3_ absorption band (11,215 eV). The Ir-L_3_ absorption line was recorded in transmission mode by using the X-ray absorption spectrometer (Rigaku R-XAS Looper, Rigaku, Tokyo, Japan, Country) [37,38]. The continuous radiation was obtained from an X-ray tube with molybdenum target and LaB_6_ filament and the monochromatic light was obtained with a curved Ge (220) single-crystal monochromator. A scintillation detector measured the transmitted intensities. The EXAFS analysis was done as a standard procedure, using the REX2000 package [39]. The refinement procedure involves the subtraction of pre-edge and post-edge backgrounds from the experimental spectra. The EXAFS χ(k) functions were calculated from the post-edge oscillations of the Ir-L3 band normalized through the smooth atomic absorption (post-edge background). The spectral conversion from the *k*-space into physical *r*-space was done with the Fourier transformed procedure. The radial functions exhibit several maxima assigned to the atomic distances around the neighboring iridium absorbing atoms. The first main maxima correspond to the first shell atoms which surround the central Ir and were isolated by Hanning-function windows. The fitting procedure gives the coordination numbers and interatomic distances between the iridium absorbing atoms and the atoms from the first and second shells of coordination. The theoretical EXAFS spectra concerning electron backscattering amplitudes, phases, and inelastic mean free path were obtained with the FEFF6 code [40]. 

HPLC spectra were recorded with a Thermo Scientific Ultimate 3000^™^ UHPLC+ (Thermo Fisher Scientific, Waltham, MA USA), with a DIONEX binary RS PUMP, RS Autosampler, RS Column Compartment equipped with RS Diode Array Detector, RS Fluorescence Detector. The equipment was fitted with a Varian Pursuit 5 C18 column (250 × 4.6 mm^2^). Samples were analyzed using an isocratic method, with acetonitrile as the eluent at 20 °C with a flow rate of 1 mL min^−1^.

NMR data were obtained using an Agilent Technologies DD2 600 MHz NMR spectrometer (Bruker BioSpin GmbH, Rheinstetten, Germany) having a 5 mm PFG Triple Resonance Cold probe at 25 °C. ^1^H and ^13^C chemical shifts (δ) are referenced to the signal of deuterated chloroform. The organometallic compound, IrQ(ppy)_2_ (0.28 wt/v %), was characterized by ^1^H, ^13^C, and ^15^N-NMR spectroscopy. The assignments of proton and carbon atoms were based on heteronuclear multiple-bond correlation spectroscopy (^1^H-^13^C-HMBC), heteronuclear single quantum coherence spectroscopy (^1^H-^13^C-HSQC), correlation spectroscopy(^1^H-^1^H−COSY), total correlation spectroscopy (^1^H-^1^H-TOCSY) and nuclear Overhauser effect spectroscopy (^1^H-^1^H-NOESY) experiments. NMR spectra were processed using M-Nova software (version 14.1.1).

The X-ray diffraction (XRD) measurements were made on IrQ(ppy)_2_ powder using a Bruker D8 Advance type X-ray diffractometer (Bruker AXS GmbH, Karlsruhe, Germany) with copper target X-ray tube and LynxEye one-dimensional detector. The X-ray tube parameters were fixed at 40 kV and 40 mA. CuK_α1_ radiation (λ = 1.54056 Å) was used as an X-ray source. Crystal Sleuth software (2006) was used for the analysis of XRD data. 

## 3. Results and Discussion

**Figure 1:**
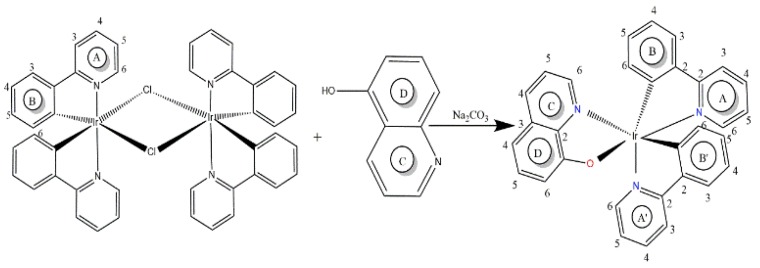
Synthesis route of IrQ(ppy)_2_.

The earlier papers containing the X-ray crystal structures of the similar organometallic compounds reported a significant *trans*-effect of the Ir-C, which induces the formation of the Ir-Cl bridge bonds trans to the Ir-C bonds. Based on this *trans*-effect, the structure illustrated in Figure 1, leads to an isomer with the C- and N-donor atoms having a *trans* position to each other [41,42,43].

The synthetic route to IrQ(ppy)_2_ implies the reaction between the [(C^N)_2_Ir-µ-Cl]_2_ dimers and 8-hydroxyquinoline ligand in the presence of Na_2_CO_3_ catalyst. In this reaction, cleavage of the chlorine bridge and binding of the quinoline ligand occurs, by N^O coordination mode.

### 3.1. EXAFS Measurements

To verify the molecular structure of IrQ(ppy)_2_, we performed the EXAFS spectroscopy to determine the Ir coordinations by using a Rigaku X-ray spectrometer.

The Ir-L_3_ EXAFS measurements allow us to accurately determine the metal environment in the Ir(III) organic complexes with efficient phosphorescence emission. Such knowledge will enable us to verify the correctness of the preparation stages of these compounds, by comparison with the molecular structure experimentally derived by XRD. 

**Figure 2:**
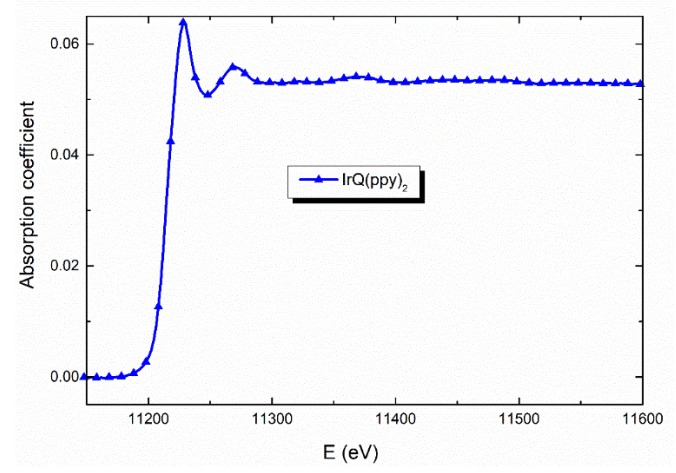
X-ray absorption spectra of the IrQ(ppy)_2_ organometallic compounds.

Figure 2 has shown the X-ray absorption coefficient for the Ir-L_3_ edge after refinement and normalization with the sample thickness. The oscillations around the Ir-L_3_ edge allows the calculations for the bonding between the metal and ligands (Ir-N, Ir-C, Ir-O), in the first and second shell but also the interatomic distances in both shells. The photoelectron transitions between Ir 2p_3/2_ and the upper states 5d_3/2,5/2_ give the line absorption for the Ir-L_3_. Between 11.20 and 11.28 keV, the line is quite intense, suggesting an efficient 2p_3/2_-5d electron transfer to Ir unoccupied.

**Figure 3:**
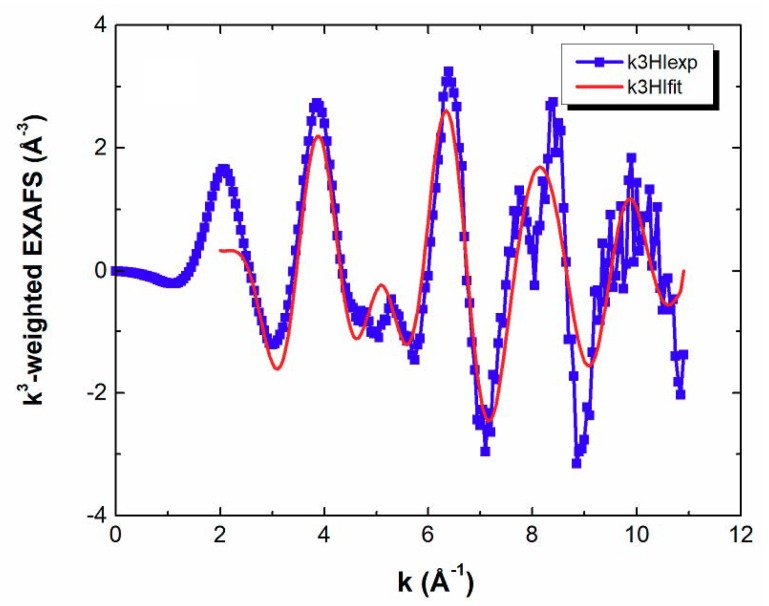
Extended X-ray absorption fine structure EXAFS k3χ(k) spectra of IrQ(ppy)_2_ experiments.

The EXAFS of IrQ(ppy)_2_ (Figure 3) has sharp and intense features for the k between 0 to 6 and weakens between 7 to 11. This fact allows better calculation for distances in the first and the second shell but with some errors for the third shell.

**Figure 4:**
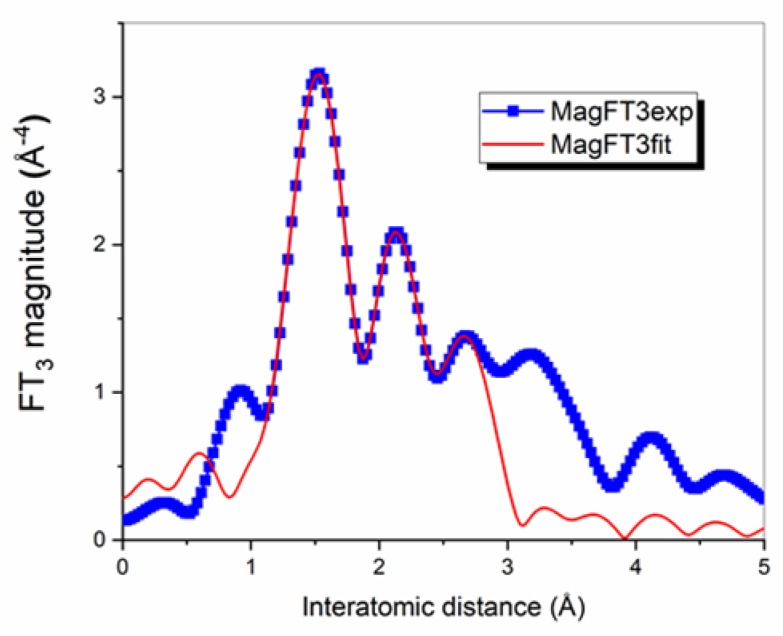
EXAFS FEFF9 calculations of IrQ(ppy)_2_.

Figure 4 shows the magnitude of the Fourier transforms of the Ir L_3_-EXAFS spectra of the intermediate and final compounds, over the k-range 2–11 Å^−1^. The results of the fit (coordination numbers and interatomic Ir-neighbour distances) were compared with molecular bonds from experimentally derived X-ray diffraction on powder, for IrQ(ppy)_2_ final compound [26].

In the IrQ(ppy)_2_ compound, the Ir environment from the XRD measurements consists of 2 C, 3 N and 1 O nearest neighbors (first shell) at the average distances 2.03, 2.08 and 2.15 Å, respectively, and 11 C next-nearest neighbors at an average distance of 2.98 Å (Table 1). EXAFS provides for this compound 4 (C, N, O) nearest neighbors at 1.97 Å and a split next-nearest neighboring C shell, with 5 C at 2.63 Å and 6 C at 2.96 Å.

The EXAFS results for the first shell indicate a mean value of 1.97 Å because all three species, C, N, O, are seen as mean elements corroborated with their coordination number and cannot be individually assigned. The mean coordination number represents a superimposed contribution with distances varying between 1.82 to 2.04 Å, which induces a decrease in the oscillations in amplitude. This fact was observed in other iridium-based compounds where the contributions of the atoms from the first shell of Ir are not resolved and give a single shell [43].

Two main discrepancies are therefore noticeable between EXAFS and the model obtained from XRD analysis: a contraction of the interatomic distances between Ir and its nearest neighbors and a split of the next-nearest neighboring C shell, for IrQ(ppy)_2_. The splitting was also observed by Feiters et al. detected by EXAFS as a weakening of contribution, and adequately simulated with a split shell, but different distances [44]. A possible explanation in our case is related to the lower electronegativity of oxygen which tightens the rings of quinoline ligand moving the carbon atoms towards iridium. Concerning the contraction of the interatomic distances between Ir and its nearest neighbors, this might be related to the π–π stacking between molecules in the crystalline structures, as can also be seen in the XRD measurements.

### 3.2. High-Performance Liquid Chromatography

The reaction between the [(C^N)2Ir-µ-Cl]_2_ intermediate dimers and 8-hydroxyquinoline (Q) was investigated by HPLC to prove the conversion from the dimer to the desired IrQ(ppy)_2_ and to analyze the purity of the end-product. Solutions of dimers, quinoline and end-product, as well as 2-phenylpyridine (ppy), in acetonitrile AcCN, were independently injected into an HPLC system equipped with a diode array detector. 

**Figure 5:**
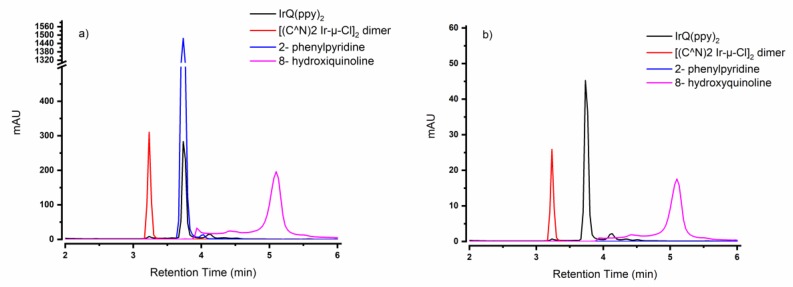
High-performance liquid chromatography (HPLC) analysis of IrQ(ppy)_2_, dimer and ligands at (**a**) 261 and (**b**) 403 nm.

The chromatograms (Figure 5a,b) obtained showed distinct retention times between the dimer (3.23 min), end-product (3.73 min) and quinoline (5.10 min). However, the retention time for 2-phenylpyridine was similar to that of the end-product (i.e., 3.73 min). To distinguish between the two peaks, the chromatograms at four distinct wavelengths (261, 340, 403 and 450 nm) were analyzed. 2-phenylpyrdine has a peak with strong absorbance at 261 nm, but the absorbance at the other three wavelengths is negligible, whereas the absorbance for IrQ(ppy)_2_ is evident at all four wavelengths. The broader absorbance spectrum is expected due to the presence of the MLCT bands. The differences in the peaks of IrQ(ppy)_2_ and 2-phenylpyridine can also be observed in the 3D field spectra. In addition to confirming the presence of distinct species and the formation of the end-product, HPLC suggested a purity of more than 95% according to peak integration.

### 3.3. Nuclear Magnetic Resonance

**Figure 6:**
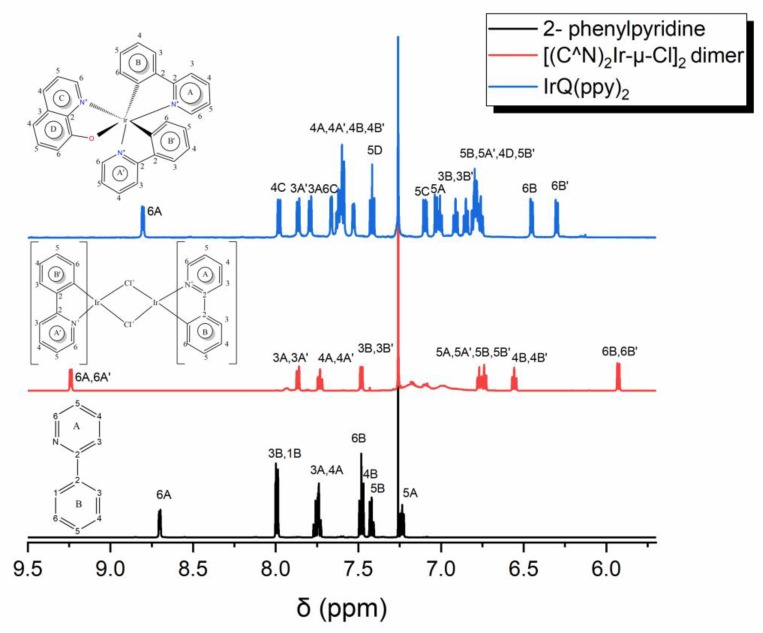
Nuclear magnetic resonance (^1^H NMR) spectra of 2-phenylpyridine, dimer and complex.

In addition to HPLC, the reaction was analyzed by ^1^H NMR and ^13^C NMR spectroscopy. The ^1^H NMR spectra of the 2-phenylpyridine ligand, [(C^N)_2_Ir-µ-Cl]_2_ dimers and IrQ(ppy)_2_ compounds (Figure 6) illustrate the presence of one compound in solution. The proton H^6B^ of the 2-phenylpyridine ligand is chemically shifted from δ 7.48 to 5.93 and 6.45 ppm, upon coordination to the metal, in comparison with the iridium dimer and IrQ(ppy)_2_, respectively, which implies the expected effect of cyclometalation at C^1B^ (C^1B′^). 

Interestingly, the most downfield proton H^6A^, neighboring the N atom, in the free 2-phenylpyridine ligand, exhibits a low-field shift from δ 8.70 ppm in the ligand to δ 9.24 ppm in the dimer, owing to deshielding by the proximal electronegative nitrogen. In the IrQ(ppy)_2_ molecules, the first proton H^6A^ shifts further (from δ 9.24 ppm in the dimer to δ 8.80 ppm in the complex) upon the cyclometalation process with the 8-hydroxyquinoline ligand. This shift can be used as a diagnostic method to prove the coordination processes [45].

Full ^1^H and ^13^C NMR spectroscopy characterization and assignment were carried out using 2D techniques including ^1^H-^1^H COSY, ^1^H-^13^C HMBC, ^1^H-^13^C HSQC, ^1^H-^1^H TOCSY and ^1^H-^1^H NOESY.

The signals in the 1D ^1^H and ^13^C NMR spectra of the IrQ(ppy)_2_ organometallic compound showed the expected number of protons: 22 protons (16 protons belonging to two phenylpyridine and six protons to quinoline ring) and the expected 31 carbon atoms (11 × 2 = 22 from phenylpyridine and nine from quinoline ligands) from which nine carbon atoms are quaternary. TOCSY spectra of the IrQ(ppy)_2_ organometallic compound show six distinct groups of protons that were assigned to the six aromatic rings in the structure. (ESI, Figure S1)

There are two groups with three protons connected through bonds only in the quinoline ligand (Table 2), therefore the groups with three protons described before come from the quinoline ligand. The complete assignment of all the quinoline protons thus requires a thorough analysis of its ^1^H-^1^H COSY and ^1^H-^1^H TOCSY spectra.

From those two groups of 3three protons (H^4C^, H^5C^, H^6C^; H^4D^, H^5D^, H^6D^), we aim to identify the location of each proton in the pyridine and benzene rings in the quinoline ligand. In the COSY spectra of pyridine and benzene rings of quinoline ligand (see ESI, Figure S2), the proton at δ 7.98 ppm shows strong interaction with a proton (δ 7.11 ppm) and weak interaction with a proton at δ 7.67 ppm. In the 1H-1H COSY spectrum (ESI, Figure S3), the proton at δ 8.80 ppm shows strong interaction with the proton at δ 7.00 ppm, which may be a vicinal coupled, and weak coupling with protons at δ 7.79 and 7.62 ppm (see ESI, Figure S3). The proton at δ 7.79 ppm shows strong coupling with the proton at δ 7.62 ppm (vicinal coupling) and weak couplings with protons at δ 8.80 and 7.00 ppm. The proton at δ 7.00 ppm shows coupling with the proton at δ = 7.62 ppm. Similarly, the right order of the protons in the next aromatic rings in phenylpyridine ligands was identified based on 1H-1H COSY (Table 3). In the ^15^N-HMBC spectrum (ESI, Figure S4), the only proton at δ 7.67 ppm shows a signal. Thus, it follows that the proton at δ 7.67 ppm could be attributed to H^6C^ because the corresponding carbon is bound to N. This proton at δ 7.67 ppm shows in the COSY spectrum a weak interaction with δ 7.98 ppm proton and we can distinguish between the signals at δ 7.11 and 7.98 ppm that correspond to H^5C^ and H^4C^ protons. The strong interaction in the COSY spectrum between the protons at δ 7.98 and δ 7.11 ppm completes this assumption. In this way, we assigned the protons from the pyridine ring of the quinoline ligand. The rest of the protons (δ 7.03, δ 6.80, and δ 7.42 ppm) could be assigned to the benzene ring. From the COSY spectrum of the benzene ring, the proton at 7.03 ppm shows strong interaction with the proton at 7.42 ppm and weak interaction with the proton at 6.80 ppm. These coupling interactions were not enough to distinguish the position of each proton in the benzene ring. 

The strong correlation between δ 6.80 and δ 7.42 ppm in the ^1^H-^1^H COSY spectrum allows the assignment of the proton at δ 7.42 ppm to H^5D^. Next, the last proton from the benzene ring at δ 7.03 ppm could be assigned to H^6D^. In the ^1^H-^1^H COSY spectrum, the proton at δ 7.03 ppm (H^6D^) shows a signal at 7.11 ppm (H^5C^), which confirms the assignments in the benzene ring (H^4D^, H^5D^, H^6D^) of the quinoline ligand. 

Regarding phenylpyridine ligand, from TOCSY maps we found four sets with four protons, as assigned in Table 3. (ESI, Figure S1).

In the ^1^H-^15^N-HMBC spectrum (ESI, Figure S4), the protons at δ 8.80 and 7.53 ppm show strong signals, which enable us to assign these protons and their groups with the pyridine ring from phenylpyridine ligand. Moreover, these protons are located proximal to the N atom in pyridine rings. Therefore, the protons at δ 8.80 and 7.53 ppm could be attributed to H^6A^ and H^6A’^. Based on previously observed correlations in ^1^H-^1^H COSY spectrum, the signals at δ 8.80, 7.00, 7.62, and 7.79 ppm can be assigned to H^6A^, H^5A^, H^4A^ and H^3A^, respectively (ESI, Figure S4).

**Figure 7:**
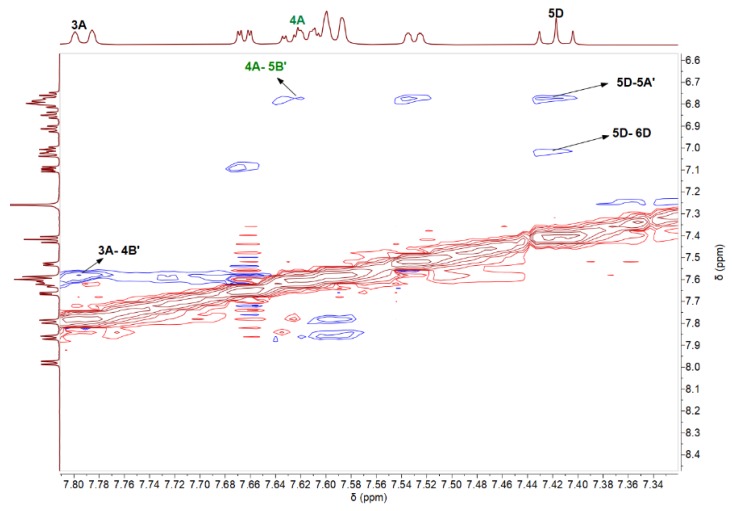
Vital NOESY correlations of IrQ(ppy)_2_.

The relative orientation of ligands was determined by acquiring the ^1^H-^1^H NOESY spectra (Figure 7). In the ^1^H-^1^H NOESY spectrum, the signals appear because the protons are close together in space and not through bonds. Proton H^5D^ (δ 7.42 ppm) from the benzene ring of quinoline ligand shows a strong correlation in the ^1^H-^1^H NOESY spectrum with H^6D^ (δ 7.03 ppm), which is its neighbor, and H^5A’^ (δ 6.79 ppm) from pyridine ring of phenylpyridine ligand. In this way, we can confirm the position of the pyridine ring from phenylpyridine ligand in the IrQ(ppy)_2_ structure. More interesting was the observation of the correlations between H^4A^ (δ 7.62 ppm) and H^5B’^ (δ 6.77 ppm) and between H^3A^ (δ 7.79 ppm) and H^4B’^ (δ 7.60 ppm), which determined the orientation of phenylpyridine ligands to each other. NOESY experiment has shown that in the structure of IrQ(ppy)_2_ molecule the pyridine ring A and phenyl ring B are tilled.

The coordination of bidentate nitrogen from the quinoline ligand to the IrQ(ppy)_2_ core preserves a *trans* configuration of the pyridine groups similar to the one in the precursor [(C^N)_2_Ir-µ-Cl]_2_ dimers and this observation have been confirmed both by ^1^H-^1^H COSY and ^1^H-^1^H NOESY experiments. These results are similar to the literature for mononuclear iridium(III) complexes displaying the IrN_4_C_2_ coordination [44]. 

Kappaun et al. described a similar compound, ppy_2_Irq, together with other quinolinolate complexes, but from the NMR measurements, the authors concluded a *cis* disposition for the two carbon ligands and the *trans* position for the 2-phenylpyridine nitrogen atoms [46]. Their organometallic molecule was not found to be luminescent, even in degassed solvents, the explanation being connected with an unfavorable mixing of singlet and triplet excited states for this compound. Our compound exhibits dual emission, green and red and similar electroluminescence with orange all over color [25].

For a correct assignment, these results might be compared with those obtained from ^1^H-^13^C-HSQC and ^1^H-^13^C-HMBC (ESI, Figures S5 and S6). The ^1^H-^13^C-HSQC experiment was used to determine chemical shifts of individual carbon atoms, which directly bound protons. All nine quaternary carbons (see Table S1, rows in red from ESI) in the IrQ(ppy)_2_ organometallic compound were assigned based on correlations in ^1^H-^13^C-HSQC (ESI, Figure S5)

These spectra reveal correlations between carbons and protons that are separated by two, three and sometimes four bonds. These results agree with the ^1^H-^1^H COSY and ^1^H-^1^H NOESY results. Briefly, in the pyridine ring of phenylpyridine ligand, correlations between the proton H^6A^ and carbons 2A, 4A, 5A can be identified. The proton H^5B^ shows correlations with carbons 4B and 3B. The proton H^5B′^ shows a correlation with quaternary carbon 1B′. In the ^1^H-^13^C-HMBC spectrum, the proton H^5D^ shows a strong correlation with carbon 1D, and proton 6A′ shows a strong correlation with the carbon 5A′. (ESI, Figure S6).

All NMR measurements reveal a *trans* isomerization of the IrQ(ppy)_2_ similar to the Ir(ppy)_3_ organometallic and the chemical yield of this compound was estimated to be 85%–90%.

### 3.4. X-ray Diffraction Analysis

The measured powder by NMR (in solutions) and EXAFS studies were subjected to X-ray diffraction analysis. After the final step of the reaction, between [(C^N)_2_Ir-µ-Cl]_2_ compound and the 8-hydroxyquinoline ligand, the solvent was eliminated with a rotary evaporator. The resulting slur was dissolved in dichloromethane to remove the catalyst and other impurities. This solution was then filtered and the IrQ(ppy)_2_ powder was obtained after recrystallization from solution. The drying procedure significantly influences the crystallography of the final compound.

**Figure 8:**
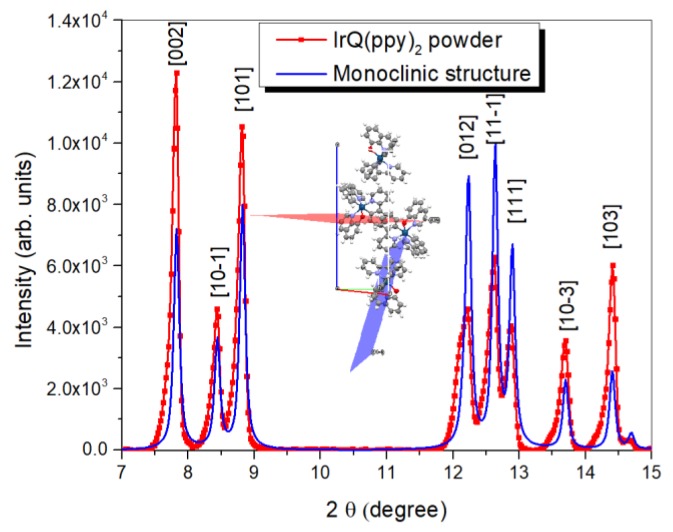
XRD patterns of the IrQ(ppy)_2_ powder.

XRD measurements revealed a monoclinic structure for the IrQ(ppy)_2_ (Figure 8) with the following parameters: a = 11.8; b = 9.7 and c = 22.9 and angles α = β = γ = 90^○^. Similarly, Chun Yi obtained a monoclinic structure (P2(1)/n) for the IrQ(ppy)_2_ with the following parameters: a = 11.5; b = 9.4 and c = 22.6 and angles α = γ = 90^○^, but with β = 93.12°, which is the angle between the basal plane and the c axis [26].

The XRD patterns show a different orientation between the monoclinic structure of IrQ(ppy)_2_ reported by Chun Yi and our sample, suggesting a faster crystallization along with the c-axis leading to needle-like structures (nanowires) and a slower one along the a and b axis.

The IrQ(ppy)_2_ powder can be easily oxidized in the atmosphere during crystallization from the solution, which significantly reduces the phosphorescent properties of this compound.

For an efficient phosphoresce, the recrystallization process was done in a vacuum at 50 °C which induces a faster growth of IrQ(ppy)_2_ nanocrystals along the c-axis. When the recrystallization is done at room temperature, the structure is almost similar to that obtained by Chun Yi [26].

In the powder form, a π–π stacking process between the adjacent IrQ(ppy)_2_ takes place and distances between quinoline ligands compete with the intermolecular interactions [18,47] which induces inter-ligand energy transfer (ILET) [48]. The emission processes follow the excitation in the ^1^MLCT state and an efficient transfer to the ^3^MLCT due to the strong spin-orbit coupling.

## 4. Conclusions

The EXAFS measurements confirm the IrQ(ppy)_2_ organometallic compound in comparison with the previous XRD data. The presence of the oxygen atoms influences the splitting of the next-nearest neighboring C in the second shell from the Ir^3+^ ions. The contraction of the interatomic distances in the first shell can be correlated with the π-π stacking between quinoline ligands during the crystallization process. The interatomic distances in the first and second shells obtained from the EXAFS measurements were compared with the *trans* structure obtained from NMR studied.

The signals in the 1D NMR spectra showed the expected number of protons: 22 protons (eight ppy H resonances twice and six quinoline H resonances). The proton signals were assigned to their position in the bicycling system of the ligand by COSY, HMBC, HSQC, TOCSY and NOESY and reveal the presence of 31 carbon atoms, from which nine carbon atoms are quaternary. All these measurements suggest the formation of the desired compound IrQ(ppy)_2_, containing two pyridine ligands in *trans* isomers with a distribution of nitrogen ions in a similar way with facial Ir(ppy)_3_. These three N ions form a triangle which can be seen in the ^15^N-HMBC and, in comparison with fac-Ir(ppy)_3_, the trans-IrQ(ppy)_2_ presents higher photoluminescence quantum efficiency compared with the equivalent cis-IrQ(ppy)_2_ and dual green-red electroluminescence. The efficiencies of chemical reaction resulting from NMR measurements for IrQ(ppy)_2_ vary between 85% and 90% for different samples, while from HPLC the chemical reaction is over 95%.

Finally, XRD measurements confirm the monoclinic structure of the obtained IrQ(ppy)_2_ powder, where the contraction of the interatomic distances between Ir and its nearest neighbors suggests a π–π stacking between quinoline ligands during the crystallization process, along the *c*-axis, due to a faster crystallization process obtained during the evaporation of the solvent.

## Figures and Tables

**Table 1:** Average coordination number obtained by EXAFS curve fitting.

Shells	Structural Model Based on XRD ^a^	EXAFS ^b^		
	N/R (Å)	N	R (Å)	σ^2^ (Å^2^) *x*10^−3^
1st shell	1 O/2.15 Å 1 N/2.13 Å 2 N/2.03 Å 2 C/2.03 Å	6 (C,N,O)	1.97 ± 0.02 Å	10 ± 3
2nd shell	11 C/2.98 Å	5 C 6 C	2.63 ± 0.02 Å 2.96 ± 0.03 Å	8 ± 3 6 ± 2

^a^—data from literature; [26] for the final compound; ^b^—our work.

**Table 2:** Quinoline protons identified from total correlation spectroscopy (TOCSY) map.

No of Sets	1 H- δ (ppm)		
5	7.98	7.67	7.11
6	7.03	6.80	7.42

**Table 3:** Phenylpyridine protons identified from TOCSY map.

No of Sets	1 H- δ (ppm)			
1	8.80	7.00	7.62	7.79
2	7.86	7.63	6.79	7.53
3	6.45	6.79	7.60	6.92
4	6.30	6.77	7.60	6.85

## Supplementary Materials

The following are available online at https://www.mdpi.com/1996-1944/13/7/1617/s1, Figure S1: TOCSY spectrum of IrQ(ppy)2. Figure S2: COSY spectrum of quinoline ligand. Figure S3: COSY spectrum of phenylpyridine ligand. Figure S4: 1H-15N HMBC of IrQ(ppy)2. Figure S5: 1H-13C HSQC spectrum of IrQ(ppy)2, Figure S6: 1H-13C HMBC spectrum of IrQ(ppy)2. Table S1: Assignments of IrQ(ppy)2 organometallic compound from 1H-NMR and 13C-NMR.
