# Supplementary material for: Detailed Molecular and Structural Analysis of Dual Emitter IrQ(ppy)2 Complex
Source: Materials (Basel). 2020 Apr 1;13(7):1617. doi: 10.3390/ma13071617 (PMC7178428; doi:10.3390/ma13071617)
Supplement: Supplementary file 1 [file materials-13-01617-s001.pdf]

materials

MDPI

Supplementary Materials: Detailed Molecular and Structural Analysis of Dual Emitter IrQ(ppy)₂ Complex

Iulia Corina Ciobotaru [1], Daniel Nicolae Crisan [1], Primoz Šket [2], Constantin Claudiu Ciobotaru [1,*] and Silviu Polosan [1,*]

[1] National Institute of Materials Physics, Atomistilor 405A, 077125 Magurele, Romania; corina.ciobotaru@infim.ro (I.C.C.); daniel.crisan@infim.ro (D.N.C.)

[2] Slovenian NMR Center, National Institute of Chemistry, Hajdrihova 19, Ljubljana, SI-1000, Slovenia; primoz.sket@ki.si

* Correspondence: claudiu.ciobotaru@infim.ro (C.C.C.); silv@infim.ro (S.P)

Figure S1 presents the Total Correlation Spectroscopy (TOCSY spectrum) of the organometallic IrQ(ppy)$_2$ compound in which the cross-peaks are observed not only for nuclei which are directly coupled but also between nuclei which are connected by a chain of couplings. For example, in the case of phenylpiridine, the proton 6A is coupled with 5A, 4A and 3A. The proton 3A′ is coupled with 5A′, 6A′ and 4A′; the proton 6B is coupled with 5B, 3B and 4B and the proton 6B′ is coupled with 5B′, 3B′ and 4B′.

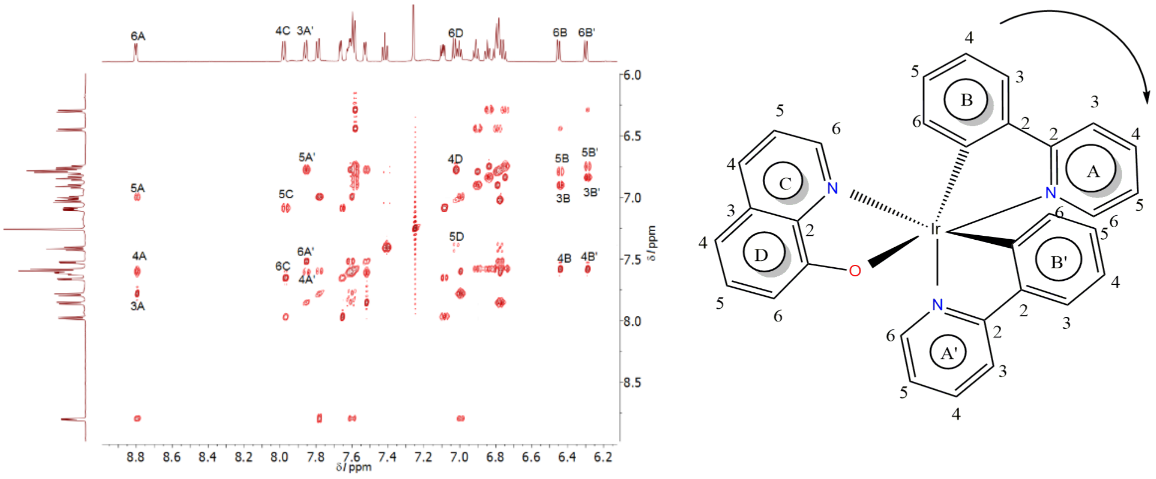

Figure S1. spectrum of IrQ(ppy)$_2$.

In the case of quinoline ligand, the proton 4C is coupled with 5C and 6C; the proton 6D is coupled with 4D and 5D. TOCSY map reveals all 6 groups of protons (2 groups with 3 protons from quinoline ligand and 4 groups with 4 protons from the phenylpiridine ligand.

Figure S2 presents Correlation Spectroscopy (COSY) for determining the connectivity between protons based on geminal and vicinal couplings for quinoline ligand. For example, the proton 4C shows strong interaction with 5C and a weaker one with 6C. The proton 6D shows strong interaction with 5D and the weaker one with 4D.

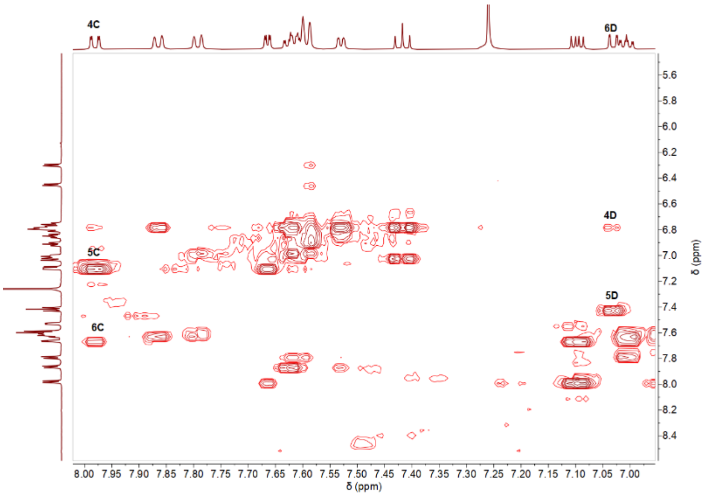

Figure S1. COSY spectrum of quinoline ligand.

Figure 3. present Correlation Spectroscopy (COSY) for phenylpiridine ligand. For example, the proton 6A shows strong interaction with 5A and a weaker one with 3A and 4A. The proton 6A′ shows strong interaction with 5A′ and the weaker one with 3A′. The proton 6B shows strong with 5B and weaker with 3 B and 4B and the proton 6 B′ show strong interaction with 5B′ and the weaker one with 3B′ and 4B′.

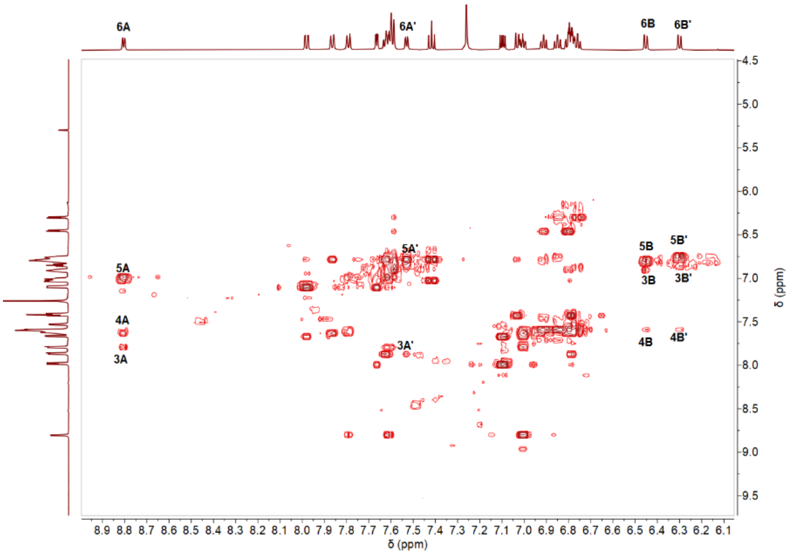

Figure S3. COSY spectrum of phenylpyridine ligand.

Figure S4 presents the ^{15}N-HMBC shows the correlations between protons and 15N nuclei. There are 3 nitrogen atoms in IrQ(ppy)$_2$. The protons 6A, 6A′ and 6C that are located proximal to the N atom in phenylpyridines and quinoline ligands, shows strong signals in the ^{15}N-HMBC spectrum.

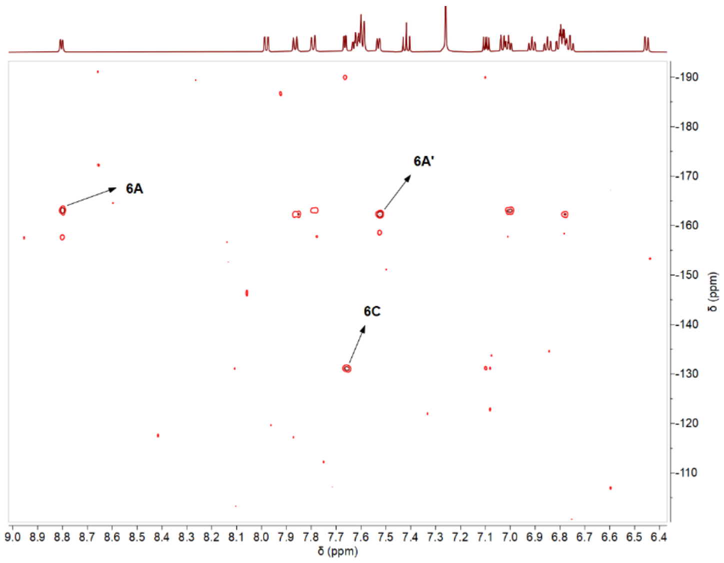

Figure S4. ^{1}H-^{15}N HMBC of IrQ(ppy)$_2$.

Figure S5 reveals the 9 quaternary carbon atoms from IrQ(ppy)$_2$ molecule by ^{1}H-^{13}C HSQC spectrum.

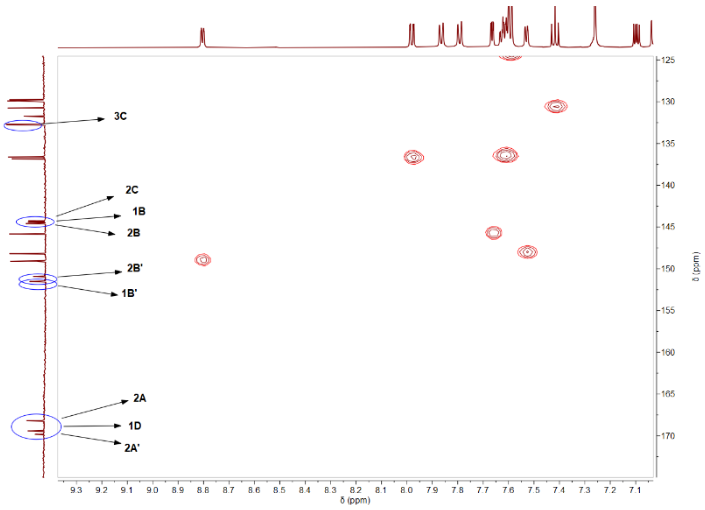

Figure S5. ^{1}H-^{13}C HSQC spectrum of IrQ(ppy)$_2$.

Figure S6 presents the correlation between carbons and protons that are separated by two, three and sometimes four bonds through ^{1}H-^{13}C HMBC analysis.

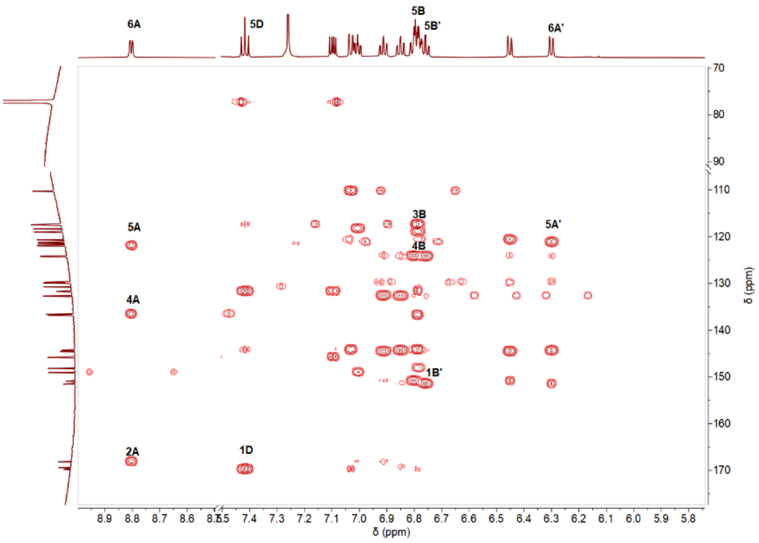

Figure S6. ^{1}H-^{13}C HMBC spectrum of IrQ(ppy)$_2$.

Table S1 evidences the molecular protons and carbons assignments in the IrQ(ppy)$_2$ molecule. Red lines show the quaternary carbons in the quinoline and phenylpiridine ligands.

Table S1. Assignments of IrQ(ppy)$_2$ organometallic compound from ^{1}H-NMR and ^{13}C-NMR.

Atom	δ ^{1}H (ppm)	δ ^{13}C (ppm)
2A		168.17
3A	7.79	118.30
4A	7.62	136.60
5A	7.00	121.88
6A	8.80	149.03
1B		144.59
2B		144.40
3B	6.92	120.64
4B	7.60	124.13
5B	6.79	129.87
6B	6.45	132.66
2A’		169.41
3A’	7.86	119.10
4A’	7.63	136.60
5A’	6.77	121.86
6A’	7.53	148.17
1 B’		151.50
2 B’		150.90
3B’	6.85	121.19
4B’	7.60	124.19
5B’	6.77	129.77
6B’	6.30	132.69
2 C-D*		144.26
3 C-D*		131.71
4C	7.98	136.79
5C	7.11	121.27
6C	7.67	145.78
1D		169.81
4D	6.80	110.22
5D	7.42	130.72
6D	7.03	121.84

*-marks the carbon atoms between phenyl and pyridine rings in quinoline ligand.
